# Pollution and Ecological Risk Evaluation of Heavy Metals in the Soil and Sediment around the HTM Tailings Pond, Northeastern China

**DOI:** 10.3390/ijerph17197072

**Published:** 2020-09-27

**Authors:** Wei Zhang, Jinghua Long, Xueru Zhang, Weining Shen, Zhongyi Wei

**Affiliations:** 1 School of Public Administration, Hebei University of Economics and Business, Shijiazhuang 050061, China; zhangdaw007@heuet.edu.cn (W.Z.); zhangxueru5@pku.edu.cn (X.Z.); shen@heuet.edu.cn (W.S.); 2 College of Land and Environment, Shenyang Agricultural University, Shenyang 110866, China

**Keywords:** heavy metal, tailings pond, sediment, potential ecological risk

## Abstract

Tailings ponds are a main heavy metal pollution source in mining areas. In this study, the geo-accumulation index ($I_{geo}$) and the potential ecological risk index (RI) are used to evaluate the environmental impact of Hongtou Mountain (HTM) tailings pond on the surrounding area. Farmland soil, surface water, and sediment samples in the Hun River around the HTM tailings pond were collected. Heavy metal contents in the samples were analyzed by Inductively Coupled Plasma Mass Spectrometry (ICP-MS). Results show that Cu, Zn, and Cd content in the farmland soil and sediment around the lower reaches of the Hun River (HTM tailings pond section) are obviously higher than the upper reaches. The $I_{geo}$ values show that the farmland soil near the outlet of the tailings pond is the most polluted area. Cu was classified as moderate–strongly pollution, Zn was moderately pollution, and Cd was strongly pollution. Cd is the major pollutant in farmland soil, the monomial ecological risk ($E_{r}^{i}$) for Cd is a very high potential ecological risk. The potential ecological risk of sediment in the dry season is more serious than in the raining season. In the dry season, the $I_{geo}$ index shows strong pollution for Cu and Cd at the confluence of the Hun River and the tributary from the HTM tailings pond, and a moderate–strongly pollution for Zn. Whereas, the $E_{r}^{i}$ index shows that the monomial ecological risk for Zn at H3 is low, and Cu is moderate. The potential ecological risk at H3 is high, and Cd is the main source of the ecological risk around the HTM tailings pond.

## 1. Introduction

Heavy metal pollution in farmland soil and sediment has become a serious environmental problem, special in the mine area. Heavy metal in farmland soil could be easily uptaken by crops, and enter the food chain, caused serious harm to human health [1,2,3]. Most of the heavy metals were accumulated in the sediment after entering the river, and heavy metals in the sediment could also release into the water, disrupt the water ecosystem [4,5].

The exploitation of mineral resources promoted economic development and also caused serious environmental problems. A large number of tailings were deposited in the tailings pond during the beneficiation process [6,7]. Tailings pond was a main pollution source in the mine area, required strict operation management [8]. Previous studies have shown that tailings pond could cause an increase of heavy metal content in the surrounding area, such as farmland soil, river, and reservoir, etc., [9,10,11,12]. Liu et al. found that Cu, Zn, and Cd were the main pollutants in the sediment around the copper mine areas [13]. Lina et al. found that higher concentrations of particulate tungsten were found in the surface water downstream of a scheelite tailings pond [14].

The oxidation of sulfide tailings was the source of the mine environmental pollution [15,16]. During the process of sulfides oxidation, acid mine drainage water generated, the following chemical reactions will occur:

4FeS_2(s)_ + 15O_2_ + 2H_2_O → 2Fe_2_(SO_4_)_3_ + 2H_2_SO_4_ (1)

Fe_1−x_S_(s)_ + (2−x/2)O_2_ + xH_2_O → (1−x)Fe^2+^ + SO_4_^2−^ + 2xH^+^ (2)

The decrease in the pH value could significantly promote the release of heavy metals from tailings [17]. A large amount of heavy metals would release from the tailings pond during the long-term oxidation process [18]. The amount of heavy metals discharged from the Laver mine (Sweden) account for approximately 4–12% of the amount annually released by sulfide oxidation [19,20]. Zhang et al. found that the oxidized zone reached to 0.4 m in Hongtou Mountain (HTM) copper mine tailings pond, and the heavy metal content in oxidized tailings was obviously lower than unoxidized tailings, approximately 762.75 t of sulfur, 6997.5 kg of Zn, and 86.06 kg of Cd are released from the HTM tailings pond to surrounding area during the last 20 years [21].

Hongtou Mountain (HTM) tailings pond located at the upper reaches of the Hun River, had a potential threat for human health. The aim of this paper was to investigate the environmental impact of HTM tailings pond on the surrounding area, and evaluate the pollution situation of soil and sediment around the HTM tailings pond.

## 2. Materials and Methods

### 2.1. Site Description

**Figure 1:**
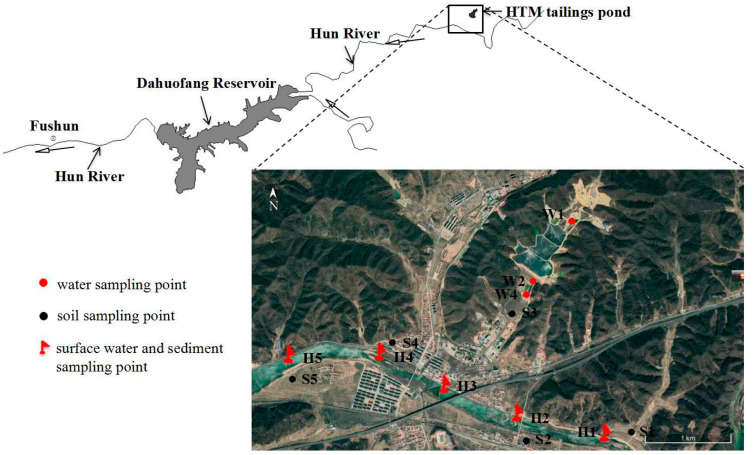
Sampling points in the research area. HTM, Hongtou Mountain.

Hun River originated in the east of the Fushun city, Liaoning province, China, stretching 415 km [1]. The Dahuofang Reservoir, located at the middle reaches of the Hun River, was the important drinking water source for Shenyang, Fushun, and Anshan city, etc., [3,5]. The Hongtou Mountain copper mine was a polymetallic mine that produced copper, zinc, gold, silver, and sulfate. The main ore minerals were pyrite (FeS2), chalcopyrite (CuFeS2), and pyrrhotite (Fe1-xS), and also contained minor quantities of chalcocite (Cu2S) and sphalerite (ZnS) [21]. HTM tailings pond located at a valley, about 2 km distance from the upper reaches of the Hun River (Figure 1).

HTM tailings pond consisted of three parts (1#, 2#, and 3# tailings pond). 1# tailings pond was located in the upper part of the valley and was still running; 2# tailings pond was located in the middle; and 3# tailings pond was located in the lower part of the valley. 2# and 3# tailings pond had abandoned for approximately 20 years [21]. Tailings were discharged with water into the tailings pond, and over 3 Mt of tailings were deposited in the HTM tailings pond. The basic physicochemical properties of tailings in 0–100 cm depth were shown in Table S1. Previous studies have shown that Cu, Zn, and Cd were the major pollutants in the tailings [21].

Normally, drainage water was discharged into the sedimentation tank; purification treatments (natural sedimentation, pH regulation, and physico-chemical purification) were conducted before recycling and discharge.

### 2.2. Sample Collection and Preparation

Water samples were collected at W1 (low-lying area in the tailings pond), W2 (low-lying area in the front of the tailings dam), W3 (surface water in 1# tailings pond), and W4 (outlet of the sedimentation tank) (Figure 1).

Soil samples were collected from different farmland (0~20 cm) around the HTM tailings pond, S2 and S4 were taken from a paddy field, S1, S3, and S5 were taken from a cornfield. Crop grains were collected near the corresponding soil sampling points.

Surface water and sediment samples in Hun River were collected at five sampling points in April (dry season) and July (raining season). Each sampling point was 1 km apart from the other. H3 was set in the confluence of the Hun River and the tributary from the HTM tailings pond. H1 and H2 were set at the upper reaches of the Hun River (HTM tailings pond section), H4, and H5 were set at the lower reaches. In the middle of the river, surface water samples were collected at 50 cm below the surface by a polyethylene container, and surface sediment samples were collected by a grab-gravity mud sampler at the same time.

Water samples were filtered with a syringe filter (0.45 μm), and acidified the samples (pH < 2) with HNO_3_ (1 mol L^−1^), stored in the fridge at 4 °C. Soil and sediment samples were air dried, and passed through a 2-mm sieve. Plant samples were washed and dried at 40 °C to a constant weight in a drying oven [22]. Each sample was conducted in triplicate.

### 2.3. Pollution and Ecological Risk Evaluation

The geo-accumulation index ($I_{geo}$) and the potential ecological risk index (RI) were used to evaluate the pollution and ecological risk of heavy metals in soil and sediment near the tailings pond.

#### 2.3.1. Geo-Accumulation Index

Geo-accumulation index ($I_{geo}$) was widely used for the heavy metal pollution assessment in soils or sediments [23,24,25]. The geo-accumulation index was calculated using the following equation [26]:

(3) $$I_{geo}=log_{2}\frac{C_{n}}{1.5BE_{n}}$$

where *C_n_* is the concentration of heavy metal in the soil or sediment; 1.5 is the modified index; *BE_n_* is the background value of the heavy metal in the soil or sediment.

The geo-accumulation index is classified into seven categories: Unpolluted ($I_{geo}$ < 0); unpolluted–moderately (0 ≤ $I_{geo}$ < 1); moderately polluted (1 ≤ $I_{geo}$ < 2); moderate–strongly polluted (2 ≤ $I_{geo}$ < 3); strongly polluted (3 ≤ $I_{geo}$ < 4); strongly–extremely polluted (4 ≤ $I_{geo}$ < 5); extremely polluted (5 ≤ $I_{geo}$) [27].

#### 2.3.2. Potential Ecological Risk Index

The potential ecological risk index (RI) was used to evaluate the risk of heavy metals in soil and sediment near the tailings pond. The potential ecological risk index considers not only the content of heavy metals, but also the ecological effect, environmental effect, and toxicological effect of heavy metals [28,29]. The potential ecological risk index RI was calculated using the following equation [30]:

(4) Eri=Tri·Cmeasured valueiCri

(5) RI=∑i=1nEri=∑i=1nTri·Cmeasured valueiCri

where RI is the potential ecological index for the sampling point; $E_{r}^{i}$ is the potential ecological risk index of a particular heavy metal *I*; $T_{r}^{i}$ is the toxic-response factor for the given heavy metal *I*; $C_{\mathrm{measured}\;\mathrm{value}}^{i}$ is the concentration of heavy metal *i* in soil or sediment; $C_{r}^{i}$ is the background value of the heavy metal *i* in soil or sediment (Table 1). The $T_{r}^{i}$ for Cu, Zn, and Cd are 5, 1, and 30, respectively.

The degree of monomial ecological risk is classified into five categories: Low potential ecological risk ($E_{r}^{i}$ < 40); moderate potential ecological risk (40 < $E_{r}^{i}$ ≤ 80); considerable potential ecological risk (80 < $E_{r}^{i}$ ≤ 160); high potential ecological risk (160 < $E_{r}^{i}$ ≤ 320); very high potential ecological risk ($E_{r}^{i}$ > 320).

The grade of the potential ecological risk index of the three heavy metals (RI): Low potential ecological risk (RI < 150); moderate potential ecological risk (150 < RI ≤ 300); high potential ecological risk (300 < RI ≤ 600); very high potential ecological risk (RI > 600).

### 2.4. Analytical Methods

Water and soil (solid-to-water ratio = 1:2.5) pH values were measured by a pH meter (PHS-3C). Soil and sediment samples were digested with the HCl-HNO_3_-HF-hClO_4_ mixed-acid digestion method. 0.5 g samples were added into a Teflon crucible (50 mL), heated on an electric hot plate, and 5 mL hydrochloric acid (HCl), 15 mL nitric acid (HNO_3_), 10 mL hydrofluoric acid (HF), and 5 mL perchloric acid (hClO_4_) were added into the crucible in sequence [31]. All the acid used in the digestion process was guaranteed reagent level (GR, 99.8%). Crop grains were digested with the HNO_3_-hClO_4_ mixed-acid digestion method, 1.0 g plant samples were added into a conical flask (150 mL), and 20 mL HNO_3_-hClO_4_ mixed-acid (4:1, *v/v* ) were added into the conical flask, and heated on an electric hot plate [22]. The concentrations of heavy metals in the extractants and water samples were measured by Inductively Coupled Plasma Mass Spectrometry (Agilent 7700× ICP-MS). A certified soil reference material (GBW07401, National Research Center for Certified Reference Materials, China) was used to ensure the accuracy of the analytical data and the accuracy ranged from 93.7 to 104.1%.

## 3. Results and Discussion

### 3.1. Heavy Metal Contents in Surface Water at the HTM Tailings Pond

As shown in Table 2, the pH value of the surface water at W1 was 4.2, the Cu, Zn, and Cd contents were 732, 566, and 7.0 µg L^−1^, respectively. The lower pH value and higher contents of heavy metals in surface water were due to the oxidation of sulfides in tailings.

The pH value of the drainage water was 4.0 at W2, the Cu, Zn, and Cd contents were 503, 550, and 4.1 µg L^−1^, respectively. The pH value of drainage water from the beneficiation process was around 7.8 to 8.1. Activated sludge process was used to decrease the heavy metal concentrations in drainage water. After water purification treatment, the pH value of drainage water decreased to 6.0, the Cu, Zn, and Cd contents in the water decreased by 55%, 46%, and 48%, respectively, compared to that of the untreated drainage water at the W3 sampling site. However, the Zn and Cd contents in drainage water still significantly higher than the threshold value in Environment quality standards for surface water of China (GB3838-2002).

### 3.2. Heavy Metal Contents in Farmland Soils and Crops around the HTM Tailings Pond

The pH values of the farmland soil around the HTM tailings pond ranged from 5.7 to 6.4, and the pH value of soil at S3 was obviously lower than that of other sampling points (Table 3).

S1 and S2 are located at the upper reaches of the Hun River (HTM tailings pond section), a region that was relatively less affected by tailings pond. The mean content of Cu, Zn, and Cd in this region was 32.2, 76.8, and 0.83 mg kg^−1^, respectively. S3 is located at the lower reaches of HTM tailings pond in a small tributary of the Hun River. Due to the direct influence of mine drainage, the mean value of Cu, Zn, and Cd at S3 was 232, 278, and 2.68 mg kg^−1^, respectively, clearly higher than that of other sampling points. S4 and S5 located at the lower reaches of the Hun River (HTM tailings pond section), and the mean contents of Cu, Zn, and Cd in this region were 76.8, 139, and 1.50 mg kg^−1^, respectively. The contents of Cu, Zn, and Cd in the downstream soil increased by 139%, 81%, and 81%, respectively, compared to the upstream region.

Heavy metal in farmland soil could be uptaken by crops, caused an increase of the heavy metal content in grain [32]. Due to the increase of Cu, Zn, and Cd content in the soil, the Cu, Zn, and Cd content in downstream crops clearly higher than at upstream. Cu content in maize at S3 and S5 increased by 92% and 17% compared to that of the S1, Zn content in maize at S3 and S5 increased by 113% and 70% compared to that of the S1, and Cd content in maize at S3 and S5 increased to 0.60 and 0.29 mg kg^−1^, significantly higher than the food safety standard for maize of China (0.1 mg kg^−1^) (GB2762-2017).

Cu and Zn content in rice at S4 increased by 108% and 104% compared to that of the S2, and Cd content in rice at S4 increased to 0.38 mg kg^−1^, significantly higher than the food safety standard for rice (0.2 mg kg^−1^) (GB2762-2017).

### 3.3. Heavy Metal Contents in the Surface Water and Sediment in HTM Tailings Pond Section of the Hun River

#### 3.3.1. Surface Water

**Figure 2:**
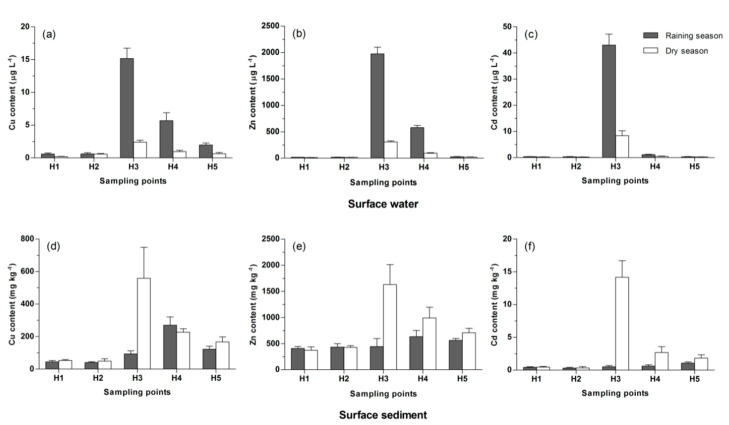
Cu, Zn, and Cd contents in the surface water and sediment of the Hunhe river. Mean values ± S.D. are shown (*n* = 3). (**a**) Cu content in surface water; (**b**) Zn content in surface water; (**c**) Cd content in surface water; (**d**) Cu content in surface sediment; (**e**) Zn content in surface sediment; (**f**) Cd content in surface sediment.

As shown in Figure 2a–c, the Cu, Zn, and Cd content in the surface water in the range of 0.2–15.2, 11.7–1974.1, and 0.3–43.1 µg L^−1^, respectively. The mean value of Cu, Zn, and Cd in the water in the raining season was clearly higher than in the dry season, which was because of the heavy metals released from tailings that could enter the river with rainfall-runoff in the raining season. Normally, the heavy metal content in surface water was higher in the dry season, and lower in the raining season. However, in this research area, a large amount of heavy metals are released from the HTM tailings pond in the raining season, caused a significant increase of heavy metal contents in surface water around the tailings pond. The effect of tailings pond on surface water was weaker in the dry season. The effect of tailings pond was the major cause of the difference between the raining and dry season.

In the raining season, the Cu, Zn, and Cd content in the surface water at H3 increased to 15.2, 1974.1, and 43.1 µg L^−1^, respectively, were clearly higher than those of at H1 and H2. The Cu, Zn, and Cd contents in surface water in the lower reaches decreased when the distance between sampling sites and HTM tailings pond increased. The Cu and Zn content in surface water at H4 decreased by 62% and 71%, respectively, compared to that of at H3, but still significantly higher than that of at H1 and H2. The Cu Zn and Cd content in the surface water at H5 decreased to an acceptable level (Cu < 10 µg L^−1^, Zn < 50 µg L^−1^, Cd < 1 µg L^−1^) (GB3838-2002).

In the dry season, the maximum of Cu, Zn, and Cd in the surface water also appeared at H3, but the maximum of Cu content did not exceed the acceptable level.

#### 3.3.2. Surface Sediment

As shown in Figure 2d–f, in the dry season, the maximum of Cu, Zn, and Cd in the surface sediment appeared at H3, were 559.3, 1631.0, and 14.2 mg kg^−1^, respectively. The Cu, Zn, and Cd contents in the surface sediment at H4 and H5 were clearly decreased compared to those of at H3, but still higher than those of at H1 and H2.

In the raining season, the maximum value of Cu and Zn content in the sediment appeared at H4, was 270.3, and 631.4 mg kg^−1^, and the maximum value of Cd content in the sediment appeared at H5, was 1.1 mg kg^−1^. The accumulation of heavy metals in the sediment resulted from the mining and acid mine drainage, and the river dilution effect decreased during the dry season. As a result, the mean value of Cu, Zn, and Cd in the sediment in the raining season were clearly lower than those in the dry season. Similar results were reported in previous studies [5,33].

### 3.4. Environmental Risk Assessment of Heavy Metals in the Soil and Sediment Around the HTM Tailings Pond

#### 3.4.1. Farmland Soil

The results of the geo-accumulation index showed that Cu and Zn at S1 and S2 were classified as unpolluted (Table 4). The $I_{geo}$ value of Cu, Zn, and Cd were highest at the S3 sampling point, Cu at S3 was classified as moderate–strongly pollution, and Zn at S3 was classified as moderate pollution. The $I_{geo}$ values of Cu at S4 and S5 ranged from 1.0 to 1.2, classified as moderate pollution, and Zn at S4 and S5 were classified as unpolluted–moderately pollution. The $I_{geo}$ values of Cd indicated that moderately to strongly pollution occurred in all the farmland soils.

The $E_{r}^{i}$ index showed that the monomial ecological risk for Cu at S3 was a moderate ecological risk (Table 4). Cd was the major pollutant in farmland soil, the $E_{r}^{i}$ values of Cd at S1, S2 and S5 were 168.9, 200.0 and 295.6, respectively, classified as high potential ecological risk, the $E_{r}^{i}$ value of Cd at S3 and S4 was 595.6 and 371.1, classified as a very high potential ecological risk.

The potential ecological risk of Cu, Zn, and Cd at S3 was obviously higher than other sampling points. Mine drainage from the tailings pond caused the increase of the Cu, Zn, and Cd content in farmland soil in the downstream area. While, for S1 and S2 point, the increase of heavy metal content in soil may is because of dust dispersion—these tiny tailings particles on the tailings pond surface could transfer to the surrounding area.

#### 3.4.2. Sediment

As shown in Table 5, the ecological risk of sediment in the raining season and dry season was obviously different, special at the H3 sampling point. In the raining season, the $I_{geo}$ index showed that the sediment at H3 was classified as unpolluted–moderately, and the $E_{r}^{i}$ and RI index showed that the potential ecological risk at H3 was low. While, in the dry season, the $I_{geo}$ index showed strong pollution for Cu and Cd at H3, and a moderate–strongly pollution for Zn.

Cd was the main source of the ecological risk in the sediment of the Hun River. The $E_{r}^{i}$ index showed that the monomial ecological risk for Cd at H3 was very high. The RI value at H3 was 468.5, and the potential ecological risk was high. The monomial ecological risk for Cd at H4 and H5 was moderate in the dry season, and obviously more serious than in the raining season. In the dry season, the heavy metals in water were retained in the sediment, lead to a serious pollution status in the sediment.

In the present study, results showed that the HTM tailings pond caused a significant increase of Cu, Zn, and Cd content in the farmland soil and Hun River near the HTM tailings pond. Heavy metals could transfer into the surrounding area by windblown and runoff. In the HTM tailings pond, remediation measures were conducted to decrease the heavy metal content in the drainage water before discharge from the tailings pond. However, results showed that Zn and Cd content in the drainage water was still obviously higher than the threshold value in Environment quality standards for surface water of China (GB3838-2002). In the raining season, most of the drainage water was directly drained off without any treatment.

Previous studies have shown that a large amounts of sulfur (S), Zn, and Cd are released from the HTM tailings pond to the surrounding area [20]. Thus, long-term and effective measures should be taken in time to prevent the discharge of heavy metals with drainage water. In addition, a surface cover system could effectively limit the process of sulfides oxidation and prevent the tiny tailings particles drifted to the surrounding area.

## 4. Conclusions

HTM tailings pond caused an increase in the heavy metal content in the farmland soil and sediment in the Hun River. The $I_{geo}$ values of Cu, Zn, and Cd were highest at S3 sampling point, Cu was classified as moderate–strongly pollution, Zn was moderately pollution, and Cd was strongly pollution. Cd was the major pollutant in farmland soil. The $E_{r}^{i}$ index showed that the monomial ecological risk for Cd at S3 was a very high potential ecological risk.

The ecological risk of sediment in the raining season and dry season was obviously different, special at the H3 sampling point. In the raining season, the $I_{geo}$ index showed that the sediment at H3 was classified as unpolluted–moderately, and the $E_{r}^{i}$ and RI index showed that the potential ecological risk in H3 was low. While, in the dry season, the $I_{geo}$ index showed strong pollution for Cu and Cd in H3, and a moderate–strongly pollution for Zn. The $E_{r}^{i}$ index showed that the monomial ecological risk for Cd in H3 was very high, and the RI value at H3 was 468.5, and the potential ecological risk was high.

In order to prevent the release of heavy metals from the HTM tailings pond with drainage water and windblown, a long-term and effective remediation strategy is necessary.

## Figures and Tables

**Table 1:** The background values of Cu, Zn, and Cd in soil, sediment, and surface water in the Hun River area.

Sample	Cu	Zn	Cd
Soil (mg kg^−1^)	24.0	62.4	0.14
Sediment (mg kg^−1^)	39.0	172	1.10
Surface water (µg L^−1^)	10.0	50.0	1.00

**Table 2:** Heavy metal contents in surface water (µg L^−1^). Mean values ± S.D. are shown (*n* = 3).

Sampling Site	pH	Cu	Zn	Cd
W1	4.2 ± 0.3	732 ± 154	566 ± 21	7.0 ± 0.4
W2	4.0 ± 0.2	503 ± 53	550 ± 29	4.1 ± 0.2
W3	7.9 ± 0.2	24 ± 8.1	481 ± 22	4.2 ± 0.4
W4	6.0 ± 0.2	10.7 ± 1.1	260 ± 19	2.2 ± 0.2
Threshold	6.0–9.0	10	50	1.0

Threshold: Environment quality standards for surface water of China (GB3838-2002).

**Table 3:** Heavy metal content in the farmland soils and crops. Mean values ± S.D. are shown (*n* = 3).

Sample Point	Soil (mg kg^−1^)				Crops (mg kg^−1^)		
	pH	Cu	Zn	Cd	Cu	Zn	Cd
S1—maize field	6.3 ± 0.3	29.8 ± 3.8	72.9 ± 6.5	0.76 ± 0.1	1.11 ± 0.1	21.2 ± 2.0	0.12 ± 0.01
S2—paddy field	6.4 ± 0.2	34.6 ± 5.1	80.7 ± 5.1	0.90 ± 0.1	1.98 ± 0.2	19.4 ± 2.1	0.08 ± 0.01
S3—maize field	5.7 ± 0.2	232 ± 27	278 ± 24	2.68 ± 0.3	2.10 ± 0.2	45.0 ± 3.4	0.60 ± 0.08
S4—paddy field	6.2 ± 0.1	80.1 ± 6.2	155 ± 21	1.67 ± 0.3	4.12 ± 0.2	39.6 ± 1.7	0.38 ± 0.03
S5—maize field	6.3 ± 0.2	72.6 ± 7.9	123 ± 9.4	1.33 ± 0.1	1.30 ± 0.1	36.1 ± 2.4	0.29 ± 0.02
Background values/threshold	-	24.00	62.40	0.14	0.20	0.20	0.10

**Table 4:** Pollution and ecological risk evaluation of heavy metals in the farmland soils around HTM tailings pond.

Sampling Points	$I_{geo}\;$			$E_{r}^{i}\;$			RI
	Cu	Zn	Cd	Cu	Zn	Cd	
S1	−0.3	−0.4	1.9	6.2	1.2	168.9	176.3
S2	−0.1	−0.2	2.2	7.2	1.3	200.0	208.5
S3	2.7	1.6	3.7	48.3	4.5	595.6	648.3
S4	1.2	0.7	3.0	16.9	2.5	371.1	390.5
S5	1.0	0.4	2.7	15.1	2.0	295.6	312.7

**Table 5:** Pollution and ecological risk evaluation of heavy metals in the sediment of the Hun River around HTM.

Sampling Time	Sampling Points	$I_{geo}$			$E_{r}^{i}$			RI
		Cu	Zn	Cd	Cu	Zn	Cd	
Raining season	H1	−0.4	0.7	−2.0	5.7	2.4	11.5	19.6
	H2	−0.53	0.7	−2.5	5.2	2.5	8.3	1.0
	H3	0.7	0.8	−1.7	12.1	2.6	13.8	28.4
	H4	2.2	1.3	−1.5	34.7	3.7	16.4	54.7
	H5	1.1	1.1	−0.6	15.7	3.3	29.5	48.4
Dry season	H1	−0.1	0.5	−1.9	6.9	2.2	11.9	21.0
	H2	−0.2	0.7	−2.3	6.3	2.5	8.9	17.7
	H3	3.3	2.7	3.1	71.7	9.5	387.3	468.5
	H4	2.0	1.9	0.7	29.1	5.7	72.9	107.7
	H5	1.5	1.5	0.2	21.4	4.1	50.0	75.6

## Supplementary Materials

The following are available online at https://www.mdpi.com/1660-4601/17/19/7072/s1, Table S1: Basic physicochemical properties of tailings in 0–100 cm depth.
