# Supplementary material for: Pollution and Ecological Risk Evaluation of Heavy Metals in the Soil and Sediment around the HTM Tailings Pond, Northeastern China
Source: Int J Environ Res Public Health. 2020 Sep 27;17(19):7072. doi: 10.3390/ijerph17197072 (PMC7578946; doi:10.3390/ijerph17197072)
Supplement: Supplementary file 1 [file ijerph-17-07072-s001.pdf]

International Journal of
Environmental Research and Public Health

MDPI

Supplementary Data

Pollution and Ecological Risk Evaluation of Heavy Metals in the Soil and Sediment around the HTM tailings pond, Northeastern China

Table S1. Basic physicochemical properties of tailings in 0-100 cm depth.

Test Items	Tailings
pH	3.2 - 4.5
Cu (mg kg^{-1})	408 - 1397
Pb (mg kg^{-1})	143 - 211
Zn (mg kg^{-1})	2985 - 5940
Cd (mg kg^{-1})	5.2 - 13.7
Cr (mg kg^{-1})	14.2 - 24.3
